# RNA sequencing and gene co-expression network of *in vitro* matured oocytes and blastocysts of buffalo

**DOI:** 10.1590/1984-3143-AR2023-0131

**Published:** 2024-06-17

**Authors:** Priscila Di Paula Bessa Santana, Kenny da Costa Pinheiro, Lino César de Souza Pereira, Soraya Silva Andrade, Flávia Figueira Aburjaile, Priscilla do Carmo de Azevedo Ramos, Eduardo Baia de Souza, Nathalia Nogueira da Costa, Marcela da Silva Cordeiro, Simone do Socorro Damasceno Santos, Moysés dos Santos Miranda, Rommel Thiago Jucá Ramos, Artur Luiz da Costa da Silva

**Affiliations:** 1 Instituto Socioambiental e dos Recursos Hídricos, Universidade Federal Rural da Amazônia, Belém, PA, Brasil; 2 Laboratório de Genômica e Bioinformática, Universidade Federal do Pará, Belém, PA, Brasil; 3 Laboratório de Fertilização In Vitro, Instituto de Ciências Biológicas, Universidade Federal do Pará, Belém, PA, Brasil; 4 Instituto Federal do Pará, Ananindeua, PA, Brasil

**Keywords:** blastocyst, buffalo, oocyte, RNA-seq, co-expression networks

## Abstract

In reproductive technologies, uncovering the molecular aspects of oocyte and embryo competence under different conditions is crucial for refining protocols and enhancing efficiency. RNA-seq generates high-throughput data and provides transcriptomes that can undergo additional computational analyses. This study presented the transcriptomic profiles of *in vitro* matured oocytes and blastocysts produced *in vitro* from buffalo crossbred (*Bubalus bubalis*), coupled with gene co-expression and module preservation analysis. Cumulus Oophorus Complexes, obtained from slaughterhouse-derived ovaries, were subjected to *in vitro* maturation to yield metaphase II oocytes (616) or followed *in vitro* fertilization and culture to yield blastocysts for sequencing (526). Oocyte maturation (72%, ±3.34 sd) and embryo development (21.3%, ±4.18 sd) rates were obtained from three *in vitro* embryo production routines following standard protocols. Sequencing of 410 metaphase II oocytes and 70 hatched blastocysts (grade 1 and 2) identified a total of 13,976 genes, with 62% being ubiquitously expressed (8,649). Among them, the differentially expressed genes (4,153) and the strongly variable genes with the higher expression (fold-change above 11) were highlighted in oocytes (*BMP15*, *UCHL1*, *WEE1*, *NLRPs, KPNA7*, *ZP2,* and *ZP4*) and blastocysts (*APOA1*, *KRT18*, *ANXA2*, *S100A14*, *SLC34A2*, *PRSS8* and *ANXA2*) as representative indicators of molecular quality. Additionally, genes exclusively found in oocytes (224) and blastocysts (2,200) with specific biological functions were identified. Gene co-expression network and module preservation analysis revealed strong preservation of functional modules related to exosome components, steroid metabolism, cell proliferation, and morphogenesis. However, cell cycle and amino acid transport modules exhibited weak preservation, which may reflect differences in embryo development kinetics and the activation of cell signaling pathways between buffalo and bovine. This comprehensive transcriptomic profile serves as a valuable resource for assessing the molecular quality of buffalo oocytes and embryos in future *in vitro* embryo production assays.

## Introduction

In buffalo, the *in vitro* production protocols often yield low rates of nuclear maturation and poor morphological quality in oocytes and blastocysts compared to other livestock animals (Di Francesco et al., 2012; Gasparrini et al., 2014; Baruselli et al., 2020; Kumar et al., 2023)⁠. Buffalo oocytes and embryos exhibit unique cellular morphology, nuclear maturation ⁠⁠(Santos et al., 2002; Neglia et al., 2003; Marin et al., 2019a), and developmental kinetics (Neglia et al., 2003; Gasparrini et al., 2014) aspects.

Adapting the *in vitro* microenvironment in a specie specific manner is essential for improving *In Vitro* Embryo Production (IVEP) performance (Lonergan et al., 2006; Marin et al., 2019b; Wang et al., 2023a). Investigating the molecular aspects of oocyte and embryo competence and understanding species-specific differences may help identify areas for protocol adaptation to enhance IVEP in a particular species.

In this context, the molecular aspects of *in vitro* maturation and embryo development in buffalo were initially explored using microarray (Kandil et al., 2010; Kumar et al., 2012; Abdoon et al., 2014) and RNA sequencing (RNA-seq) approaches (Strazzullo et al., 2014; Sood et al., 2019; Capra et al., 2020; Capra et al., 2022; Goel et al., 2022). RNA-seq estimates gene expression by quantifying the number of reads derived from each gene (Mortazavi et al., 2008)⁠⁠⁠. This method has been used to study oocyte competence (Feuerstein et al., 2012; Bunel et al., 2015; Luo et al., 2016; Du et al., 2018; Wang et al., 2023b)⁠⁠⁠⁠⁠ and embryo competence (Bauer et al., 2010; Redel et al., 2012; Cao et al., 2014; Kropp and Khatib, 2015a, 2015b; Milazzotto et al., 2016; Boroviak et al., 2018; Sood et al.; 2019; Li et al., 2020; Goel et al., 2022)⁠⁠ across various species. Moreover, to investigate *in vitro* maturation (Reyes et al., 2015; Gilchrist et al., 2016; Angel-Velez et al., 2023)⁠⁠⁠ and *in vitro* embryo development⁠⁠ in mammalian (Østrup et al., 2013; Xue et al., 2013; Graf et al., 2014; Jiang et al., 2015; Song et al., 2022; Rabaglino et al., 2023)⁠.

This study describes the transcriptomic profile of *in vitro* matured oocytes and *in vitro* produced blastocysts of buffaloes through RNA-seq, gene co-expression networks, and module preservation analysis allowing for a comprehensive comparison with the bovine transcriptomes.

## Methods

### Ethics Committee and *In Vitro* Embryo Production (IVEP)

The Ethics Committee of the Federal University of Pará (CEUA/UFPA, 2024) determined that approval was not required for samples obtained from deceased animals. Ovaries were sourced from a government-approved slaughterhouse in accordance with established procedures. The processing of samples for IVEP was conducted following ethical considerations and procedural guidelines. Each biological replicate sequenced in this study was obtained from three IVEP routines.

Buffalo crossbred ovaries were transported in 0.9% sodium chloride solution within a two-hours timeframe at room temperature. In the laboratory, follicular fluid was aspirated from antral follicles (2-8 mm diameter) using a syringe attached to an 18Ga needle. A total of 1142 *Cumulus Oophorus* *Complexes* (COCs) displaying homogeneous cytoplasm and three or more layers of compact cumulus cells were selected (Leibfried and First, 1979), and *in vitro* matured according to Da Costa et al. (2016). From the cohort that underwent *in vitro* maturation for first polar body evaluation (n=616), 410 were considered metaphase II oocytes (72%, ±3.34 s.d.). Zona pellucida was removed with 1.5 mg/ml pronase (Merck KGaA, Darmstadt, Germany), and MII oocytes were stored in RNAlater® solution (Ambion®, Thermo Fisher Scientific Inc., Waltham, MA) at -80°C until mRNA isolation (See Supplementary Figure 1a).

A total of 526 COCs *in vitro* matured oocytes followed *in vitro* fertilization and embryo culture. Frozen semen from a proven fertility buffalo underwent processing with a discontinuous density gradient Percoll column (GE Healthcare Bio-Sciences, Uppsala, Sweden), and *in vitro* fertilization according to Parrish et al. (1988). After 24 hours, presumptive zygotes were incubated in a cumulus cell monolayer in 100-µL droplets of synthetic oviductal fluid (SOF) medium with modifications (Holm et al. 1999). Drops were overlaid with sterile mineral oil and incubated at 38.5°C in a 5% CO2, 20% O2, and 75% N2 atmosphere in humidified air. Blastocyst development was assessed on the 7^th^ day (21.3%, ±4.18 s.d., n=110 blastocysts). Seventy hatched blastocysts of grade 1 and 2 quality, meeting International Society of Embryo Transfers criteria, were selected based on aspects like spherical form, the well-defined blastocele, inner cell mass, and absence of zona pellucida (Stringfellow and Seidel, 1998). These hatched blastocysts were stored in RNAlater® solution at -80°C until the mRNA isolation (See Supplementary Figure 1b).

### Library Preparation, Sequencing, and Data analysis

Two biological replicates each comprising pools of 205 metaphase II oocytes and 35 hatched blastocysts were sequenced. mRNA isolation was performed using Dynabeads© mRNA Direct Micro Kit (Life Technologies, Carlsbad, CA, USA) and single-end barcoded libraries were prepared with the Ion Total RNA-Seq Kit v2 (Life Technologies) following the manufacturer’s instructions. Each library underwent amplification, quantification on Qubit® 2.0 Fluorometer (Life Technologies) and further sequenced on the Ion Proton^TM^ System (Life Technologies).

RNA-seq data underwent trimming and filtering with a minimum PHRED quality score threshold of 20, using the FASTX-Toolkit (Hanon Laboratory, 2010), and visualization was performed with the FastQC tool (Babraham Bioinformatics, 2016). The torrent mapping alignment program (TMAP, Life Technologies) was employed to map the reads to the *Bos taurus* reference genome assembly (Bos_taurus.UMD3.1, Ensemble, release 87), allowing for two mismatches with default parameters. The combination of Burrows-Wheeler Aligner (BWA), Sequence Search and Alignment by Hashing Algorithm (SSAHA), and Super-maximal exact matches (SMEM) algorithm was configured using the “mapall” function to obtain optimal alignments (Torrent Suit Software, 2016). Mapping and coverage were visualized using *CLC Genomics Workbench 4.7.2* software (QIAGEN Bioinformatics, Aarhus, Denmark). All RNA-seq data generated in this study have been deposited, and links to the BioProject accession number PRJNA832476 can be found in the DDBJ BioProject database (NIH, 2022).

### Gene Expression and GO Enrichment Analysis

To determine the total number of genes and perform Gene Ontology (GO) enrichment analysis, each biological replicate was analyzed individually with Cufflinks (Trapnell et al., 2010)⁠⁠ for estimating relative transcript abundance. Default parameters and the *Bos taurus* UMD3.1 reference genome were used. Assemblies of each replicate were merged into the *merged.gtf* file using the Cuffmerge tool. Subsequently, the Cuffdiff tool was run using *merged.gtf*. Read counts were normalized using the Reads Per Kilobase Million (RPKM) method from the *gene_exp_diff* file, and genes with RPKM > 0.4 were considered expressed (Ramsköld et al., 2009)⁠⁠. Differentially expressed genes (DEG) were determined using HTSeq Count with *union* mode for read counting (Anders et al., 2015). Normalization and testing for differential expression were performed using the DESeq2 package (Bioconductor, 2001), based on the negative binomial distribution (Love et al., 2014)⁠⁠. The false discovery rate was adjusted to 0.05, and genes with an adjusted p-value ≤ 0.05 were considered as differentially expressed (Benjamini and Hochberg, 1995)⁠⁠⁠⁠.

Similarity analysis among samples was based on the Euclidean distance calculation, and hierarchical gene cluster analysis was generated using *regularized logarithm* transformation. Coding DNA Sequences (CDS) were obtained through the BioMart tool in the Ensembl database for enriched gene ontology categories. CDS data were uploaded to the GO FEAT tool, a free web platform (GO FEAT, 2017), which attributes functional annotation based on sequence homology with data in NCBI, Kegg, InterPro, Uniprot, Pfam and SEED databases (Araujo et al., 2018)⁠⁠.

### Preservation Module Statistics to compare Buffalo and Bovine Transcriptomic Profiles

The RNA-seq data of bovine was retrieved from the GEO platform (accession number GSE52415), selected based on the similarity of IVEP conditions with the present study (Graf et al., 2014). Data preprocessing involved aligning both buffalo and bovine transcriptomes using the same reference genome and normalizing expression data using the VST method. The treated data were used to build independent co-expression networks for buffalo and bovine in the WGCNA package within the R program (Langfelder and Horvath, 2008). Briefly, adjacency matrices were built with a soft threshold of 20, and these matrices were employed to calculate the similarity between co-expression forces, resulting in a topological overlap matrix. The Dynamic Hybrid Tree Cut algorithm delineated the branches of the clustering tree, that means the co-expression modules. Eigengene modules, representing the main components of each module, were then used to quantify the similarity between the expression profiles of the modules. Modules with very similar expression profiles (correlation of 0.75, default value) were joined and represented in a dendrogram. The co-expression networks underwent analysis for the correlation of the eigengenes modules, with those exhibiting a correlation greater than 0.9 and p-value < 0.05 considered specific stages.

To access the preservation of buffalo co-expression modules in bovine, the *modulePreservation* function of the WGCNA package was performed (Langfelder et al., 2011). The Z-summary value, indicating module preservation, was calculated, where a Z-summary value > 10 denotes strong preservation, Z-summary value between 2 and 10 indicates moderate preservation, and Z-summary < 2 denotes poor preservation. Gene ontology of the co-expression modules was performed using GO.db and AnnotationDBI packages (Bioconductor, 2001) within the R program.

## Results

### General Characterization of Transcriptome Profiles in Buffalo’s Oocytes and Blastocysts

**Figure 1:**
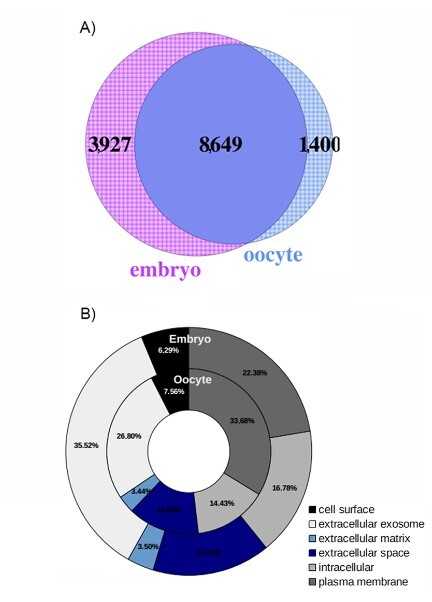
General characterization of transcriptome profiles of buffalo *in vitro* matured oocytes and blastocysts. (A) Venn diagram depicting all expressed genes (RPKM > 0.4). The intersection represents the ubiquitously expressed genes, while cracked areas denote unique genes for oocytes and blastocysts; (B) Classification of protein coding genes in embryos and oocytes based on cellular component ontology at level 4.

From the total sequenced reads for oocytes (8,014,809) and blastocysts (27,902,704), approximately 90% (7,252,174 and 24,321,010, respectively) were mapped to the reference genome. Altogether, oocytes and blastocysts expressed 13,976 genes, representing 63% of the bovine genome (22,000 genes) and the estimated buffalo genome (Rehman et al., 2021)⁠⁠⁠. Separately, oocytes expressed a total of 12,576 genes, and blastocysts a total of 10,049 genes. Of these, 62% (8,649) were ubiquitously expressed between oocytes and blastocysts (Figure 1A).

Ubiquitously genes accounted for 86% of all genes expressed in oocytes (10,049), and 68,7% of all genes in blastocysts (12,576). These genes were classified as protein coding genes (94.17%), non-coding RNAs (1.75%), pseudogenes (3.76%) and new transcripts like (0.32%). Protein coding genes ubiquitously expressed (8,144) were mainly associated with intracellular components (32.48%), plasma membrane (28.36%), nuclei (13.7%), extracellular exosome components (10.14%), and mitochondrial, Golgi apparatus, endoplasmic reticulum and extracellular components (15%). These protein coding genes were related to 362 biological functions, with 20% (1,729 genes) dedicated to cell maintenance functions such as translation, transcription, intracellular protein transport, signal transduction pathways mediated by GTPase, apoptosis regulation, cytoskeletal organization, DNA repair, replication, and chromatin remodeling. Moreover, blastocysts exhibited an abundance of non-coding RNAs (4.1%) compared to oocytes (1.72%), which may be related to their higher transcriptional activity.

### Characterization of genes exclusively expressed in oocytes and blastocysts

Exclusively expressed or unique genes are particularly significant for specific biological functions within a certain cell type (Figure 1A). Oocytes demonstrated 1,400 unique genes, constituting 14% of all expressed genes (10,049), while blastocysts exhibited 3,927 unique genes, representing 32% of all expressed genes (12,576). The subsequent analysis focused on unique genes related with specific biological functions.

In oocytes, 224 unique genes were identified, contributing to 11 biological functions related to embryo development (*SLC18A2*, SOX*, *CDKN1C*), cellular differentiation (*CCDC88A*, *SFRP1*, *MEF2C*), regulation of signaling cascades such as JAK-STAT (FLRT*) and MAPK (*PELI2*), and regulation of transmembrane transport (*CFTR*, *AKAP6*, GABR*, *GRIN2A*, *SORT1*).

In contrast, blastocysts exhibited 2,200 unique genes across 107 biological functions. These functions encompassed RNA and protein processing, differentiation, cellular proliferation, embryo development, signaling pathways such as TGFβ and BMP, fatty acids and lipids metabolic pathways, and regulation of cytokines (See Supplementary Tables). The cellular component ontology analysis supported these biological functions, revealing that 33% of oocyte-unique genes encoded proteins located in the plasma membrane, suggesting roles in signaling and transmembrane transport. Conversely, 35% of embryo-unique genes were related to exosome-contained proteins, indicating cell-signaling activity and the exchange of molecules between embryoblasts and/or extracellular media (Figure 1B).

### Characterization of Differentially Expressed Genes (DEG) and strongly variable genes

Among the ubiquitous genes, 4,153 were identified as Differentially Expressed Genes (DEG), with 3,309 being induced and 844 repressed between buffalo oocytes and blastocysts. These DEG were related to 200 biological functions, including gene expression regulation, intracellular transport of proteins, signal transduction pathways, and cytoskeletal organization. The dissimilarity between oocytes and blastocysts was evident in the Euclidean distance map, resulting in the clustering of them into separate groups, highlighting their distinct expression profiles.

**Figure 2:**
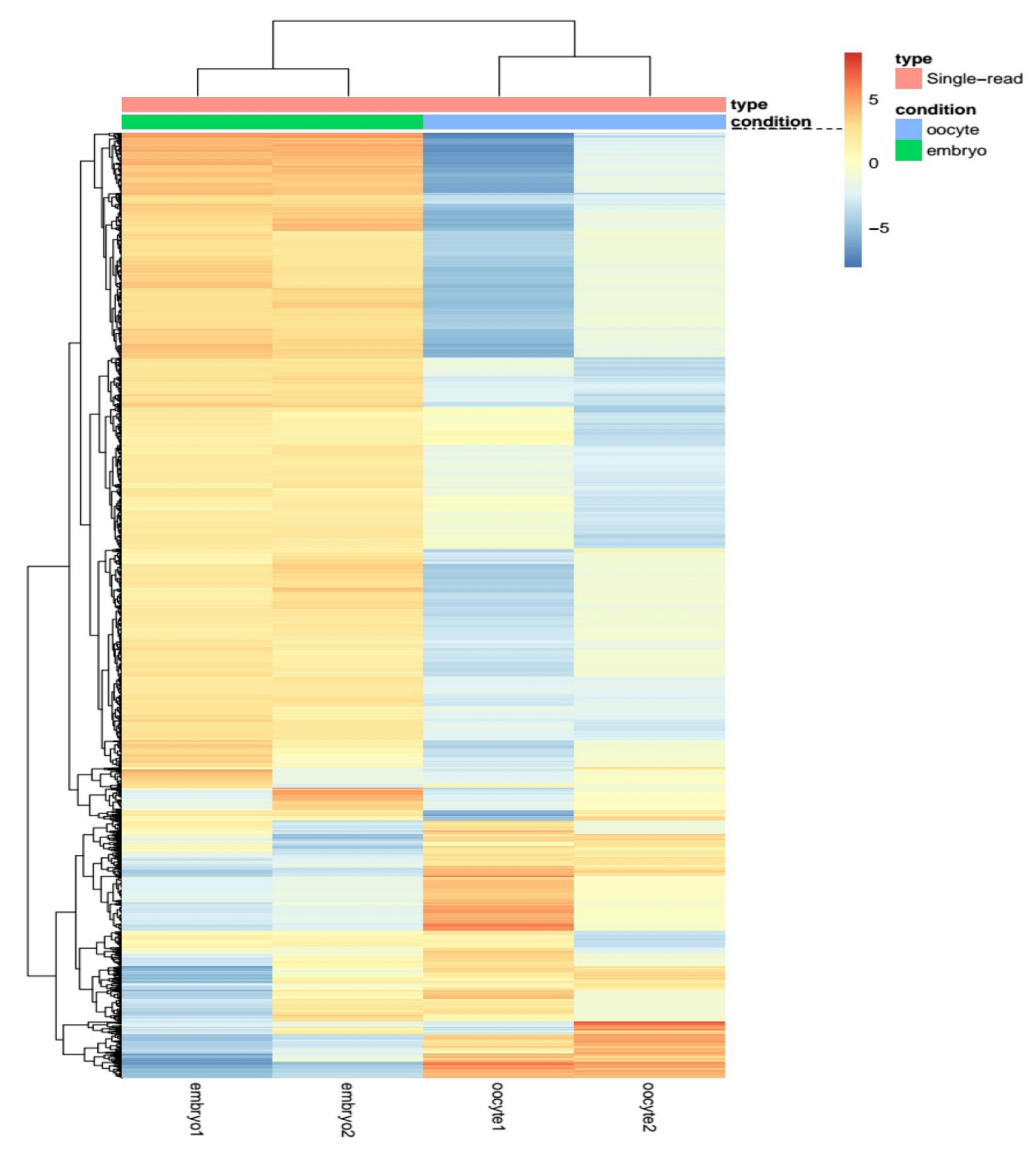
Hierarchical cluster and Heatmap of Differentially Expressed Genes (DEG). Oocytes and embryos are grouped into two clusters (n=4,153), with strongly variable genes showing a fold-change above 11.

The analysis also identified the strongly variable genes among the DEG. Using Hierarchical Cluster and Heatmap analysis (Figure 2), genes with the highest fold-change (above 11) were selected and categorized into two groups: Group 1, comprising genes highly induced in embryos and repressed in oocytes, and Group 2, including genes highly induced in oocytes and repressed in embryos (Table 1).

**Table 1:** Molecular and biological functions of the strongly variable genes.

**GROUP 1 – Induced in *in vitro* Blastocysts**			
	**Gene_symbol or Gene_id**	**Molecular function**	**Biological function**
1	ANXA2, ANXA6, DSC2, S100A14	calcium ion binding	homophilic cell adhesion via plasma membrane adhesion molecules
2	KRT18, AHNAK	RNA binding	negative regulation of the apoptotic process, regulation of RNA splicing
3	ENSBTAT00000022731.4, ENSBTAT00000022269.3	phosphatase activity	regulation of phosphatase activity
4	APOA1	cholesterol transporter activity	glucocorticoid metabolic process, integrin-mediated signaling pathway
5	APOA1	high-density lipoprotein particle binding	lipoprotein biosynthetic process, high-density lipoprotein particle assembly
6	ANXA6	ligand-gated ion channel activity	apoptotic signaling pathway, negative regulation of sequestering of calcium ion
7	ANXA2	phospholipase inhibitor activity	phospholipase inhibitor activity
8	PRSS8	serine-type endopeptidase activity	positive regulation of sodium ion transport
9	SLC34A2	transmembrane transporter activity	In utero embryonic development
**GROUP2 – Induced in *in vitro* matured Oocytes**			
	**Gene_symbol or Gene_id**	**Molecular function**	**Biological function**
1	WEE2, ATP10D	magnesium ion binding	mitotic cell cycle, negative regulation of cyclin-dependent
2	KPNA7	nuclear localization sequence binding	NLS-bearing protein import into the nucleus
3	ENSBTAT00000034504.3	ribonuclease activity	regulation of RNA stability
4	ENSBTAT00000034504.3	telomeric RNA binding	telomere maintenance via telomerase
5	UCHL1	thiol-dependent ubiquitin-specific protease activity	ubiquitin-dependent protein catabolic, negative regulation of MAP kinase activity process
6	BMP15	transforming growth factor beta receptor binding	BMP signaling pathway, granulosa cell development
7	ENSBTAT00000000819.5	translation factor activity, RNA binding	negative regulation of cytoplasmic translation
8	ENSBTAT00000065334.1	transcription factor activity, sequence-specific DNA binding	regulation of transcription, DNA-templated
9	NLRP14, NLRP8, WEE2	ATP binding	Spermatogenesis, negative regulation of cyclin-dependent protein serine/threonine kinase activity
10	ZP2, ZP4	-	binding of sperm to zona pellucida

### Comparison of gene co-expression networks of buffalo and bovine

**Figure 3:**
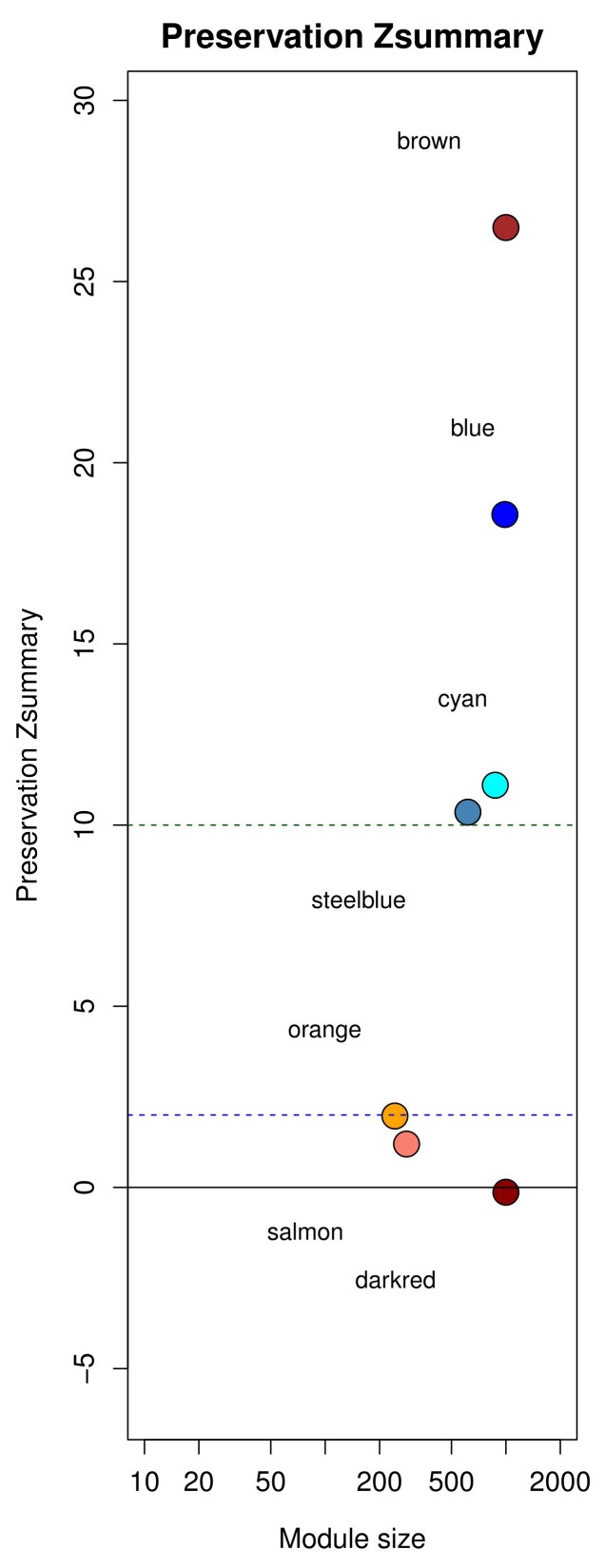
Preservation of buffalo co-expression modules in bovine and Gene Ontology. The brown (exosome component), blue (steroid metabolism), cyan (cell proliferation), and steelblue (morphogenesis) modules exhibited Z-summary > 10, indicating strong preservation. The orange and dark red (cell cycle) and salmon (amino acids transport) modules show Z-summary < 2, indicating weak preservation. Gene ontology results are provided for each module.

No specific modules were identified for buffaolo oocytes (r >0.9, p<0.05). However, for blastocysts, seven modules of co-expressed genes were identified in buffalos, with four modules showing strong preservation (Zsummary > 10) and three modules showing weak preservation (Z-summary < 2) in the bovine counterpart. According to gene ontology, the modules strongly preserved in bovine counterparts were related to exosome components, steroid metabolism, cell proliferation, and morphogenesis. In contrast, the weakly preserved modules were linked to the cell cycle and amino acid transport (Figure 3).

## Discussion

This study delves into the transcriptomic profiles of buffalo oocytes and *in vitro* produced blastocysts. The total number of genes expressed in buffalo aligns closely with previous reports in cattle, ranging from 10,494 to 13,327 genes in *in vitro* matured oocytes (Graf et al., 2014)⁠⁠⁠⁠, and from 11,501 to 13,724 genes in blastocysts (Chitwood et al., 2013; Graf et al., 2014)⁠⁠⁠⁠. Remarkably, oocytes and blastocysts collectively express around half of the buffalo genome (Rehman et al., 2021)⁠⁠⁠, and mirrors previous RNA-seq results in cattle⁠⁠⁠⁠, humans, and mice (Xue et al., 2013; Jiang et al., 2014)⁠⁠. The overlap in expressed genes between oocytes and embryos, encompassing 62%, primarily revolves around cellular maintenance functions. This concurs with existing report indicating that tissues from humans and mice might share around 75% of mRNAs encoding proteins despite their diverse functional roles (Ramskold et al., 2009).

### *In Vitro* maturation related genes expressed in buffalo oocytes

Buffalo oocytes exhibit gene expression linked to plasma membrane functions, encompassing ligand-dependent receptors for estrogen (*SFRP1*) and gamma-aminobutyric acid (*GABR*), protein transport channels (*SORT1*), amino acids (*GLRA3*), cholesterol (*CFTR*) and calcium (*AKAP6*, *GRIN2A*). Notably, genes related to the cell cycle regulation through MAP kinase (*UCHL1*) and cyclins (*WEE2*, *NLRPs*), which promote the maintenance of oocyte arrest until fertilization and are correlated with oocyte's competence (Tripathi et al., 2010)⁠. Also, transcripts for transcriptional regulation, translation, and RNA stability were strongly induced been identified as ribonuclease, telomeric, translation, and transcription factors. These transcripts may be related to the regulation of the mRNA storage in oocytes which are known to trigger early embryonic development mechanisms (Tadros and Lipshitz, 2009; Labrecque and Sirard, 2014)⁠.

Another genes related to cell signaling (*BMP15*), cell cycle (*UCHL1, WEE1, NLRPs*), RNA stability regulation (KPNA7, ENSBTAT*), and fertilization (*ZP2, ZP4*) were strongly induced in buffalo oocytes otherwise repressed in blastocysts. Likewise, karyopherins were highly expressed in *in vitro* matured oocytes and gradually decreased until the blastocyst stage in pigs. *KPNA7* gene encodes a receptor for translocation through nuclear pores and the inhibition of its translation by interference RNA in oocytes decreased blastocyst formation in pigs, thus indicating its role in oocyte competence and embryonic development (Wang et al., 2012)⁠⁠⁠⁠. *BMP15*, a growth factor, influences granulosa cells, promoting oocyte maturation (Macaulay et al., 2016) and its supplementation in maturation media increased blastocyst formation in cattle (Sudiman et al., 2014)⁠⁠⁠. While *ZP2* and *ZP4* play a crucial role in sperm binding to zona pellucida and fertilization (Yanagimachi, 1981). Their expression increases along the oogenesis and has been correlated with the oocyte morphological quality (Canosa et al., 2017)⁠⁠⁠.

### Development related genes expressed in buffalo blastocysts

Buffalo blastocysts expressed genes related to cell signaling such as Bmp (*FAM83G*, *TGFB3*, *RGMB*, *NODAL*, *RGMA*, *DSG4*, *MAPK3*, *MEGF8*, *GDF7*), the transforming growth factor beta (TGF-β) superfamily, Wnt (*WNT6*, *WNT11*, *WNT5A*) and Notch pathways (*NOV*, *PDCD10*, *SLC35C2*, *ZMIZ1*). These pathways play pivotal roles in regulating proliferation, stem cell maintenance, differentiation, and morphogenesis, influencing lineage decisions in the blastocyst (Bernatik et al., 2017; Menchero et al., 2017)⁠⁠. The *LRP5* encodes an LDL receptor in the Wnt pathway, while *NODAL* is a member of the TGF-β superfamily, both genes contribute to inner cell mass and epiblast development (Granier et al., 2011; Tribulo et al., 2017)⁠⁠, moreover embryos that failed to express them do not progress after gastrula, resulting in fetal death in mice (Conlon et al., 1994; Kelly et al., 2004)⁠⁠.⁠

Proliferation-related genes are usually linked to metabolic regulation, ensuring the production of macromolecules and metabolic energy before mitosis (Vander Heiden et al., 2009)⁠⁠⁠⁠. Buffalo blastocysts expressed the mTOR complex activator (*LAMTOR1*) also an amino-acid carrier (*SLC34A2*) which activate the mTOR (mammalian target of rapamycin) signaling pathway (Rebsamen et al., 2015)⁠⁠. mTOR induces aerobic glycolysis and increases the uptake of nutrients resulting in proliferative behavior (Murakami et al., 2004; Redel et al., 2015; Spate et al., 2015)⁠⁠. Lipid metabolism genes, including leptin transmembrane receptors (*LEP*), low-density lipoproteins (*LRP5*), and enzymes for fatty acid modification (*FA2H*) and oxidation (*ACOT8*) were also expressed. Notably, the *APOA1* gene was strongly induced in buffalo and encodes an apolipoprotein-A1 major component of high-density lipoprotein. The knockdown of APOA1 was correlated with fewer implantation sites in mice females (Jia et al., 2016)⁠⁠⁠.

Buffalo blastocysts also expressed genes related to interferon-γ and interleukin production (*RHGEF2*, *CD226*, *PRKD2, MAVS*), secretion (*FAR4*, *LRRC32*, *RGCC*), embryo development and implantation (*KRT18*, *ANXA2*, *S100A14*, *SLC34A2*, *PRSS8*, ANXA2, ENSBTAT*). Studies using RNA interference to disrupt keratin 18 (*KRT18)*, the cell adhesion molecule annexin A2 (*ANXA2),* and metalloproteinase (*S100A14)* mechanisms were detrimental to blastocyst formation in bovine (Goossens et al., 2010) and decreased the number of *in vivo* implantation sites in mice (Wang et al., 2015). *S100A14*, *ANXA2*, serine protease (*PRSS8*), and amino acid transmembrane transport (*SLC34A2*) were previously reported to play a role in implantation (Shibasaki et al., 2009; Ruan et al., 2012; Wang et al., 2015)⁠⁠. In mice, embryos secreted the serine protease trypsin that triggered cell signaling and decidualization in endometrial cells (Ruan et al., 2012)⁠⁠⁠⁠. *ANXA2* interacts with *S100A14* creating a protein complex, which may facilitate cell adhesion interactions for implantation (Myrvang et al., 2013)⁠. These genes may be related to the mechanism of implantation in buffalo, as blastocysts interact with endometrium⁠⁠⁠ cells through the secretion of signal molecules, regulating implantation and conceptus development (Bazer, 2013).

### Comparison of gene co-expression networks of *In Vitro* blastocysts of buffalo and bovine

Herein, gene co-expression networks and preserved modules analysis were employed to compare buffalo and bovine, particularly their gene co-expression relations. This methodology, previously applied in pre-implantation embryos of human, mice, bovine, marmoset, and goats (Xue et al., 2013; Jiang et al., 2014; Boroviak et al., 2018; Li et al., 2020), highlighted evolutionarily conservation in the embryonic development program across mammalian. Buffalo and bovine blastocysts exhibited a strong correlation in co-expression modules related to exosome components, steroid metabolism, cell proliferation, and morphogenesis. This suggests that these cellular functions are orchestrated by well-preserved clusters of genes, interacting in a co-expression network during embryo development.

The strong preservation of the exosome component module, implicated in immune stimulation and embryo implantation (Chen et al., 2022), underscores its crucial role in both buffalo and bovine blastocysts. Furthermore, the strong preservation of cell proliferation, morphogenesis, and steroid metabolism modules aligns with their correlation to embryo formation and tissue differentiation (Basson, 2012), also cell growth and division as steroid biosynthesis is essential for generating new cell membranes (Singh et al., 2013).

However, poor preservation of certain modules indicates differential co-expression relations during embryo development. For instance, the cell cycle module was poorly preserved, potentially explaining differences in the kinetics of embryo development between buffalo and bovine (Gasparrini et al., 2014). Similarly, the amino acid transport module, critical for cell homeostasis (Zhang et al., 2017) and signaling pathway activation (Kim et al., 2011; Rebsamen et al., 2015; Redel et al., 2015).

## Conclusion

In conclusion, this study provides a comprehensive transcriptome profile of *in vitro* matured oocytes and blastocysts from buffaloes. Prominent candidates for *in vitro* oocyte competence include *BMP15, UCHL1, WEE1, NLRPs, KPNA7, ZP2,* and *ZP4*. Similarly, genes *KRT18*, *ANXA2*, *S100A14*, *SLC34A2*, *PRSS8*, *ANXA2, LRP5*, *NODAL*, *MEGF8, LAMTOR1*, *APOA1,* *LEP,* and *ANXA6* emerge as potential candidates for *in vitro* embryo competence. The strong preservation of gene co-expression networks in blastocysts suggests a similarity in embryonic development programs between buffalo and bovine species.

## Supplementary Material

Supplementary material accompanies this paper.

Supplementary Table 1. Identification of unique genes with specific biological functions in *in vitro* matured buffalo.

Supplementary Table 2. Identification of unique genes with specific biological functions in buffalo *in vitro* produced blastocysts.

Supplementary Figure 1. Morphological quality of metaphase II oocytes and hatched blastocysts produced *in vitro*. (A) Metaphase II oocytes after removal of cumulus cells and zona pellucida. The first polar bodies visible in the photo were indicated by the black arrows. (B) A droplet from the *in vitro* culture dish on the 7^th^ day of embryo development, showing hatched blastocysts.

This material is available as part of the online article from https://doi.org/10.1590/1984-3143-AR2023-0131
