# Supplementary material for: RNA sequencing and gene co-expression network of in vitro matured oocytes and blastocysts of buffalo
Source: Anim Reprod. 2024 Jun 17;21(2):e20230131. doi: 10.1590/1984-3143-AR2023-0131 (PMC11192227; doi:10.1590/1984-3143-AR2023-0131)

Supplementary Table 1: Identification of unique genes with specific biological functions in *in vitro* matured buffalo.

*Gene Ontology – Biological Fuctions	**Gene Names
**Signaling Pathway**	
gamma-aminobutyric acid signaling pathway	GABRA3, GABRB3, GABRG1, GABRA4
fibroblast growth factor receptor signaling pathway	FGF16, FLRT2, FLRT1
neuropeptide signaling pathway	SORT1, GPR19, GLRA3
Toll signaling pathway	PELI2, PELI3, PALM3
cellular response to cAMP	RAPGEF3, CFTR, AKAP6
response to amphetamine	GRIN2A, SLC18A2, ADORA2A
**Development and Differentiation**	
neuronal action potential	SCN9A, SCN4A, SCN1A, SCN2A, SCN10A
excitatory postsynaptic potential	SEZ6, MEF2C, DGKI
neuron maturation	IRX5, FEV, CDKN1C
positive regulation of chondrocyte differentiation	ZBTB16, SOX5, SOX6
regulation of neuron projection development	FRMD7, CCDC88A, SFRP1

* Gene ontologies correspond to GO level 6, and terms with fewer than 3 hits are not shown.

** Gene without gene_symbol available were identified by ensembl_id.

Supplementary Table 2: Identification of unique genes with specific biological functions in buffalo *in vitro* produced blastocysts.

*Gene Ontology – Biological Fuctions	**Gene Names
**Cytokine and inflamatory response**	
positive regulation of interferon-gamma production	PDE4B, CD226, WNT5A, ENSBTAG00000020535
positive regulation of interleukin-6 production	ARHGEF2, WNT5A, ENSBTAG00000020535, DDX58
cellular response to cytokine stimulus	MME, FOXH1, DPYSL3
negative regulation of cytokine secretion	FFAR4, LRRC32, RGCC
positive regulation of inflammatory response	TRPV4, WNT5A, GPRC5B
positive regulation of interleukin-8 production	MAVS, PRKD2, DDX58
positive regulation of macrophage chemotaxis	TRPV4, RARRES2, MAPK3
**Differentiation, Development and Proliferation**	
negative regulation of fat cell differentiation	SIRT2, ZADH2, ZFP36L2, JDP2, WNT5A, ZFPM1, TGFB1, GPER1
endodermal cell differentiation	COL4A2, MMP14, ITGA5, COL6A1, NODAL, MMP2, COL7A1
fat cell differentiation	FFAR4, INHBB, PRLH, TTC8, SDF4, ENSBTAG00000020147, STEAP4
hematopoietic progenitor cell differentiation	DHTKD1, ENSBTAG00000005475, AGPAT5, PDGFRA, ZNF784, TGFB1
keratinocyte differentiation	ST14, WNT5A, DSG4, SCEL, DSP
melanocyte differentiation	HPS6, GLI3, EDN3, HPS4

negative regulation of osteoblast differentiation	SEMA4D, LRP5, CHRD, HAND2
chondrocyte differentiation	NOV, GLI2, TGFB1
negative regulation of chondrocyte differentiation	NKX3-2, GLI2, ADAMTS7
embryonic skeletal system development	NKX3-2, WNT5A, SULF1, PCSK5
negative regulation of neuron projection development	APOE, DPYSL3, LPAR1, ENSBTAG00000039129
keratinocyte development	EXPH5, BCL11B, KRT2
proximal/distal pattern formation	HOXC10, GLI1, HOXC11, GLI2, GLI3
negative regulation of endothelial cell proliferation	APOE, RGCC, VASH1, SULF1
positive regulation of smooth muscle cell proliferation	SULF1, NOTCH3, PDGFRB
ventricular cardiac muscle tissue morphogenesis	ENSBTAG00000040053, PROX1, SMAD7, MYBPC3, TGFB1, MYH7
branching morphogenesis of an epithelial tube	MMP14, GLI2, GDF7
cardiac muscle tissue morphogenesis	ACTC1, TBX20, ZFPM1
embryonic digestive tract morphogenesis	PDGFRA, OVOL2, GLI3
epithelial to mesenchymal transition	WNT5A, LOXL3, LOXL3, TGFB1
cell migration involved in gastrulation	LRP5, NODAL, MEGF8
gastrulation with mouth forming second	LRP5, CHRD, ZBTB17
G1/S transition of mitotic cell cycle	CCNE2, CDKN1A, CDK3, CDKN3

G2/M transition of mitotic cell cycle	CHEK2, CDKN1A, BRSK2
mitotic spindle assembly	TUBGCP6, CHEK2, MZT1
**Metabolic and biosynthetic Process**	
cholesterol metabolic process	LRP5, CLN6, LEP, CH25H, ANGPTL3, LCAT
fatty acid metabolic process	FA2H, ENSBTAG00000010270, ENSBTAG00000009788, NDUFS6, ANGPTL3
phospholipid metabolic process	PLPPR5, ENSBTAG00000008584, PLPP3, PLPP1, PLA2G5
acyl-CoA metabolic process	ENSBTAG00000010270, ENSBTAG00000009788, ACOT8
bile acid metabolic process	LEP, NR5A2, AMACR
oligosaccharide metabolic process	MOGS, ENSBTAG00000017656, ST6GALNAC2
cellular response to amino acid stimulus	LAMTOR1, PDGFRA, COL6A1, RRAGB, MMP2
response to insulin	TRPV4, LEP, PRLH, CRY2
glycogen biosynthetic process	PHKG2, AGL, PHKG1
fatty acid biosynthetic process	ENSBTAG00000004248, FA2H, ENSBTAG00000045728, ENSBTAG00000047957
positive regulation of collagen biosynthetic process	TGFB3, RGCC, F2R, TGFB1
dolichol-linked oligosaccharide biosynthetic process	ENSBTAG00000004586, PQLC3, ALG12
heparan sulfate proteoglycan biosynthetic process	EXTL1, EXTL2, B3GAT3
hydroxylysine biosynthetic process	PLOD3, PLOD1, PLOD2

**mRNA and Protein Processing**	
translation	ENSBTAG00000024125, PDF, ENSBTAG00000001794, ENSBTAG00000002565, ENSBTAG00000027930, ENSBTAG00000002330, RPL37A, RPL10L, MRPL35, METTL17, ENSBTAG00000030490, ENSBTAG00000006963, ENSBTAG00000011704, ENSBTAG00000010680, ENSBTAG00000013866, ENSBTAG00000012898, ENSBTAG00000014518, ENSBTAG00000007394, ENSBTAG00000014449, RPL17, MRPS18C, ENSBTAG00000019701, ENSBTAG00000030199, , ENSBTAG00000034503, ENSBTAG00000022534, ENSBTAG00000027015, ENSBTAG00000030185, ENSBTAG00000019007, ENSBTAG00000046949, ENSBTAG00000040435, , MRPL36, , MRPS18A, ENSBTAG00000046820, ENSBTAG00000047858, ENSBTAG00000037991, ENSBTAG00000038027, ENSBTAG00000047226
protein processing	PCSK4, CASP7, ENSBTAG00000010057, ENSBTAG00000010828, ENSBTAG00000013055, CPM, PSEN2, FKRP, IFT172, GLI3, ENSBTAG00000018000, RHBDL2
proteolysis involved in cellular protein catabolic process	CTSL, SCPEP1, CTSO, CTSF, CASP8, ADAMTS7, CTSK
positive regulation of protein catabolic process	LPCAT1, TNFSF12, RHBDD3, WNT5A, RILP
establishment of protein localization to plasma membrane	ROCK2, EFR3B, TTC8, CDH2
positive regulation of protein binding	PLXND1, CTHRC1, DACT1, WNT5A
regulation of proteolysis	LTBP4, SPOPL, ABTB1, BTBD1
rRNA processing	WDR36, ENSBTAG00000014449, ENSBTAG00000016510, DIS3L2
positive regulation of proteolysis	AURKAIP1, CLN6, FGFR4

protein N-linked glycosylation via asparagine	ENSBTAG00000017656, ST6GALNAC2, DERL3
protein O-linked mannosylation	TMEM5, LARGE2, FKRP
RNA processing	LARP6, TRUB2, DHX34
RNA secondary structure unwinding	AGO4, DDX51, ENSBTAG00000048317
**Signaling Pathway**	
BMP signaling pathway	FAM83G, TGFB3, RGMB, NODAL, RGMA, DSG4, MAPK3, MEGF8, ENSBTAG00000046807, GDF7
transforming growth factor beta receptor signaling pathway	LTBP4, FOXH1, TGFB3, SRC, DUSP15, SMAD7, PML, ZYX, ENSBTAG00000039513, TGFB1
extrinsic apoptotic signaling pathway	SGPP1, TNFSF12, SIVA1, PML, TNFRSF1B, TGFB1
phospholipase C-activating G-protein coupled receptor signaling pathway	GRPR, P2RY2, F2R, CYSLTR2, ADRA1B
protein kinase B signaling	ILK, SIRT2, RPS6KB2, GAS6, TGFB1
extrinsic apoptotic signaling pathway in absence of ligand	BAK1, BAD, ERBB3, BCL2L11
lipopolysaccharide-mediated signaling pathway	LYN, NOS3, MAPK3, TGFB1
positive regulation of apoptotic signaling pathway	ENSBTAG00000002534, PRKCD, MAGED1, INHBB
positive regulation of Notch signaling pathway	NOV, PDCD10, SLC35C2, ZMIZ1
Wnt signaling pathway	WNT6, WNT11, WNT5A, ENSBTAG00000038204
negative regulation of fibroblast growth factor receptor signaling pathway	PRDM14, WNT5A, SULF1

negative regulation of Wnt signaling pathway	BARX1, GSC, NKD2
positive regulation of Wnt signaling pathway	DACT1, ATP6V1C2, SULF1
regulation of MAPK cascade	GDF11, TGFB3, NODAL, ENSBTAG00000046807, GDF7
regulation of GTPase activity	PLXND1, SYDE2, ENSBTAG00000008858, PLXNB1, EPHA5, ENSBTAG00000020535
cellular response to growth factor stimulus	INSR, NOS1, CPNE3, GAS6
positive regulation of phosphatidylinositol 3-kinase activity	LYN, TGFB1, PDGFRB

* * Gene ontologies correspond to GO level 6, and terms with fewer than 3 hits are not shown.

** Gene without gene_symbol available were identified by ensembl_id.

Supplementary Figure 1. Morphological quality of metaphase II oocytes and hatched blastocysts produced *in vitro*. (A) Metaphase II oocytes after removal of cumulus cells and zona pellucida. The first polar bodies visible in the photo were indicated by the black arrows. (B) A droplet from the *in vitro* culture dish on the 7th day of embryo development, showing hatched blastocysts.

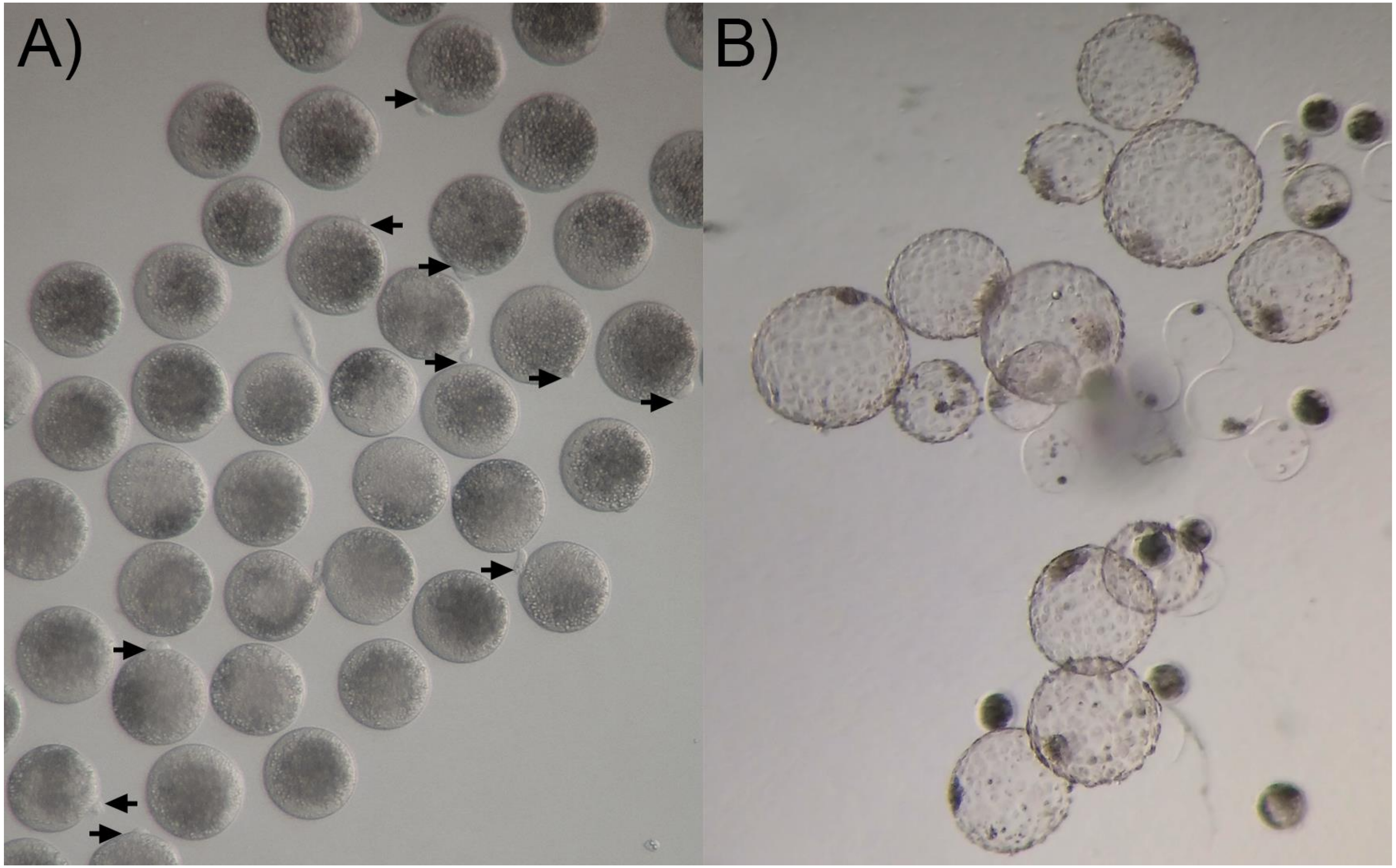

Supplement: Supplementary Table 1. [file 1984-3143-ar-21-2-e20230131-suppl.pdf]
